# Novel intraoperative strategies for enhancing tumor control: Future directions

**DOI:** 10.1093/neuonc/noac090

**Published:** 2022-11-02

**Authors:** Alexander F Haddad, Manish K Aghi, Nicholas Butowski

**Affiliations:** Department of Neurological Surgery, University of California, San Francisco, San Francisco, California, USA; Department of Neurological Surgery, University of California, San Francisco, San Francisco, California, USA; Department of Neurological Surgery, University of California, San Francisco, San Francisco, California, USA

**Keywords:** CED, glioma, glioblastoma, iMRI, surgery

## Abstract

Maximal safe surgical resection plays a key role in the care of patients with gliomas. A range of technologies have been developed to aid surgeons in distinguishing tumor from normal tissue, with the goal of increasing tumor resection and limiting postoperative neurological deficits. Technologies that are currently being investigated to aid in improving tumor control include intraoperative imaging modalities, fluorescent tumor makers, intraoperative cell and molecular profiling of tumors, improved microscopic imaging, intraoperative mapping, augmented and virtual reality, intraoperative drug and radiation delivery, and ablative technologies. In this review, we summarize the aforementioned advancements in neurosurgical oncology and implications for improving patient outcomes.

Maximal safe surgical resection is a cornerstone of treatment for patients with low- and high-grade gliomas.^1,2^ As described in previous sections of this supplement, a variety of technologies exist to enhance a surgeon’s ability to resect as much tumor as possible while limiting injury to surrounding normal tissue. In this article, we discuss novel intraoperative technologies that are under development, which aid in distinguishing tumor from normal brain with the goal of greater tumor resections.

## Novel Technologies for Intraoperative Tumor Control

### Intraoperative MRI

Magnetic resonance imaging (MRI) has long been used in the preoperative evaluation of patients with brain tumors, providing valuable insight into tumor characteristics and proximity to eloquent structures. Similarly, it has been used in the postoperative setting to evaluate tumor extent of resection (EOR). Intraoperative MRI (iMRI) for the evaluation of tumor EOR in the operating room was originally developed in the 1990s.^3^ iMRI can provide updated information on tumor location as a case progresses thereby accounting for potential increasing brain shift.

There are few randomized controlled trials (RCTs) evaluating the use of iMRI in glioma surgery. A systematic review of the literature in 2021 by Fountain et al. identified two RCTs assessing the efficacy of iMRI.^4^ One RCT included in their review was an interim analysis and included only 14 patients, finding no benefit to iMRI use.^5^ The second RCT by Senft et al. included 58 patients with known or suspected glioma with contrast enhancement on MRI. They found that patients who received iMRI were significantly more likely to undergo complete resection of their tumor, with no difference in the rate of new postoperative neurological deficits.^6^ A subsequent meta-analysis by Golub et al. used seven studies, with 236 iMRI cases and 233 conventional navigation cases, to compare iMRI to conventional navigation; iMRI was associated with increased incidence of gross total resection (GTR, OR 4.99, 95% CI 2.65–9.39, p < 0.001).^7^ Finally, a recent retrospective analysis by Shah et al. of 640 patients with supratentorial glioblastoma, 332 of whom were treated with iMRI, found iMRI to be independently associated with GTR.^8^ Overall, these studies indicate that iMRI may meaningfully contribute to increased EOR of gliomas, although some practical limitations include the availability of the technology and optimizing its use in the operating room. Interestingly, the combination of iMRI with 5-aminolevulinic acid (5-ALA) for intraoperative tumor visualization may also be beneficial, although available data in the literature is mixed and of meager quality, making this an area in need of additional investigation.^9–12^

### Intraoperative Ultrasound

In contrast to the relatively high costs associated with iMRI, intraoperative ultrasound (ioUS) is a less expensive, more accessible technology to aid in intraoperative tumor identification and resection. However, limitations of traditional ioUS include its relatively low spatial resolution and poor image quality. A meta-analysis assessing the efficacy of ioUS by Mahboob et al. found an estimated GTR rate of 77% in cases where ioUS was used.^13^ Similarly, an RCT of ioUS demonstrated a higher incidence of GTR in patients with presumed GBM where ioUS was used.^14^ Novel improved ultrasound modalities are further increasing the utility of intraoperative ultrasound for surgeons. Advanced ultrasound imaging techniques, such as navigated ioUS,^15^ contrast-enhanced ultrasound,^16,17^ and intraoperative strain elastosonography^18^ have shown promise in glioma surgery and are interesting avenues of further development. Finally, the use of contrast-enhanced ultrasound in combination with 5-aminolevulinic acid (5-ALA) has been shown to increase EOR in a retrospective study of 230 GBM patients by Della Pepa et al., highlighting the synergy of these two technologies.^19^ Overall, these various ultrasound-based technologies are limited in part by the availability of the technologies, but warrant additional investigation given their potential ability to improve EOR.

### ALA and Alternatives

5-ALA is the primary drug used for fluorescence-guided surgery in GBM. Stummer et al. demonstrated the efficacy of 5-ALA in increasing GTR and 6-month progression-free survival (PFS) in a phase III trial of high-grade glioma patients, supporting its continued use.^20^ Briefly, limitations of 5-ALA include its limited utility in low-grade gliomas as well as its sensitivity and specificity for infiltrating tumor cells.^21–23^ Additional potential alternatives for the intraoperative visualization of tumor cells include indocyanine green^24^ and fluorescein (which can also be used in combination with 5-ALA^25,26^), neither of which are molecularly tumor-specific. Tumor-specific fluorescent tags include labeled antibodies against EGFR,^27,28^ labeled chlorotoxin (which binds to a number of solid tumors),^29^ and labeled proteoglycan glypican-1 (GPC-1) antibodies.^30^ Preclinical experiments using triple-modality magnetic resonance imaging–photoacoustic imaging–Raman imaging nanoparticles have also shown promise, with highly specific accumulation in tumor relative to normal brain, highlighting the efficacy of this technology.^31^ Fluorescence lifetime imaging (FLIM), which uses time-gated intensified cameras, can be used to view nicotinamide adenine dinucleotide (NADH, which is more highly expressed in tumor relative to normal brain) and/or 5‐ALA induced protoporphyrin IX (PPIX).^32^ Studies have demonstrated the use of FLIM to highlight areas of weak 5-ALA fluorescence^33,34^ and to increase the ability to differentiate tumor from normal brain when used to detect NADH in addition to PPIX.^35^

### Intraoperative Mapping

Intraoperative mapping is frequently used for gliomas near functional brain areas and plays a significant role in maximizing safe resection.^36,37^ Indeed, a meta-analysis by De Witt Hamer et al. of 90 studies, including 8,091 patients, demonstrated reduced neurological deficits and an increased incidence of GTRs in patients who underwent intraoperative stimulation mapping when compared to those who did not.^38^ Intraoperative mapping has been combined with iMRI in a number of small studies and has been shown to be safe, albeit with little robust evidence around efficacy or impact on EOR.^39–42^ Similarly, 5-ALA has been used in combination with intraoperative mapping, with some evidence suggesting a potential benefit of using the two technologies together, but with conclusions limited by the lack of larger-sized studies.^43,44^

Intraoperative mapping techniques also continue to develop and improve. A recent report by Gogos et al. described favorable outcomes associated with asleep triple motor mapping in which bipolar and monopolar stimulation, as well as transcranial or transcortical motor evoked potentials (MEPs), are utilized to preserve cortical and subcortical motor systems during tumor resection.^36^ Similarly, a manuscript by Bander et al. highlighted the utility of high-frequency bipolar train-of-five (TOF) stimulation that combines the reliability and low incidence of intraoperative seizures seen with monopolar TOF stimulation with the focus of traditional low-frequency bipolar stimulation. Intraoperative mapping will undoubtably continue to become safer and more accurate as mapping techniques and technologies further develop.

### Intraoperative Cell and Molecular Profiling

Various intraoperative cell and molecular profiling techniques have been investigated to aid in identifying and characterizing tumor cells. Flow cytometry utilizes lasers to measure various characteristics of cells and fluorescent antibody tags; it is commonly utilized in hematologic malignancies and can be performed relatively quickly. Two recent studies have evaluated the use of intraoperative flow cytometry in tumors. Shioyama et al. describe a 10-minute protocol with the ability to calculate the malignant index (MI) of analyzed cells. They demonstrated a significant difference in MI between normal tissue and tumor, highlighting the ability to use their 10-minute protocol to quickly determine surgical margins.^45^ Alexiou et al. published a similar protocol with the ability to differentiate cell cycle of tumor cells, providing insight into tumor grade as well as information regarding surgical margins.^46,47^ A more recent study by Saito et al. also hinted at the potential utility of MI from intraoperative flow cytometry as a prognostic tool for patients with glioblastoma.^48^

Mass spectrometry (MS) is an additional technology with potential utility in the intraoperative setting. MS allows for the detailed analysis of the various lipids and proteins in a tissue sample. Desorption electrospray ionization (DESI) MS primarily provides information regarding the lipid composition of a tissue; it has been used for the intraoperative molecular diagnosis of brain tumors and to determine tumor margins, correlating well with traditional histopathological analysis.^49–51^ DESI MS has also been utilized to identify tumoral onco-metabolites. Santagata et al. demonstrated the ability for DESI MS to identify 2-hydroxyglutarate (2-HG), a downstream byproduct of mutated isocitrate dehydrogenase 1 and 2 (IDH 1 and 2), providing rapid intraoperative information on the IDH mutational status of a tumor, as well as further contributing to the determination of tumor margins.^52^ Intraoperative determination of IDH status may play an important role in decision making around EOR, as previous studies have implicated certain IDH1 mutant tumors as more amenable to maximal surgical resection.^53^ Indeed, given the potential utility of this information, PCR based platforms have also been developed for the intraoperative determination of IDH and telomerase reverse transcriptase promoter (TERTp) mutations.^54–56^

Interestingly, there is evidence that tumor specimens obtained by a cavitron ultrasonic surgical aspirator (CUSA) can be used successfully for flow cytometry and other cellular analyses.^57,58^ As technology continues to improve, it is possible that rapid flow cytometry or DESI MS will be performed directly from CUSA specimens, educating a surgeon not only on tumor margins, but, in the case of flow cytometry, potentially leveraging the range of antibodies available to yield additional information on the cellular composition of the tumor microenvironment. As we continue to move towards increased precision medicine approaches for GBM, insights from intraoperative flow cytometry or DESI MS could also inform surgical decision making and the intraoperative delivery of targeted therapies.

### Exoscopes

Several different technologies have attempted to utilize robotics to improve and enhance glioma surgery. While many of these are in their infancy, one of the most well-described is the use of exoscope platforms. Exoscopes are digital microscopes that are fixed above the surgical field and allow a surgeon to view the surgical site on a screen, rather than looking through a lens, as in a traditional microscope.^59^ Primary advantages of exoscopes relative to traditional operating microscopes are: higher magnification of the surgical field, increased tissue clarity, a larger field of view, improved ergonomics, and the potential ability to view the field in 3D or using augmented reality.^59–61^ Both exoscopes and microscopes can allow for results from preoperative Diffusion Tensor Imaging (DTI) and fiber tractography to be overlaid onto real-time images of the brain intraoperatively, potentially improving the safety of resections.^61^ Despite these theoretical benefits, few studies have assessed the efficacy of exoscopes in glioma surgery. A recent study by Baron et al. including 26 GBM patients who underwent surgical resection with the use of a 2D exoscope, demonstrated a median EOR of 94.8%.^61^ Additional larger studies directly comparing exoscopes to traditional operating microscopes are needed to further assess the impact of exoscopes on EOR, postoperative deficits, survival, and surgeon comfort in glioma surgery. Research efforts are also being directed at the combination of exoscopes with 5-ALA, which may potentially enhance the efficacy of both technologies.^62–64^ Practical limitations of exoscopes include the learning curve associated with their use and concerns surrounding depth perception with 2D exoscopes.^65^

### Raman-Based Intraoperative Tools

Raman-based intraoperative imaging methods have been increasingly studied within brain tumor surgery and seek to bring detailed information regarding tumor infiltration and pathology to the operating room.^66–68^ The two most well-described Raman-based methods in glioma surgery to this point include Stimulated Raman scattering (SRS) microscopy and Raman spectroscopy.

SRS microscopy was originally described in 2008 and allows for the rapid label and processing free analysis of biological tissues.^69^ Ji et al. subsequently demonstrated the potential promise of this technology in preclinical brain tumor models, highlighting the ability of SRS microscopy to accurately identify tumor margins.^70^ These results were followed by a study in human samples, which similarly showed that SRS microscopy was highly sensitive and specific for tumor infiltration.^71^ To take SRS microscopy directly to the operating room, Orringer et al. then developed a portable SRS microscopy system as well as stimulated Raman histology (SRH) which generated highly accurate virtual hematoxylin and eosin-stained slides.^72^ Most recently, SRH has been used in combination with deep convolutional neural networks to provide a brain tumor diagnosis in under 150 seconds; this was shown to be noninferior to pathologist interpretation of histologic images in a multicenter clinical trial including 278 patients.^73^ SRH is undoubtably an exciting tool with the ability to provide surgeons with rapid and accurate information on tumor margins and classifications.

In contrast to SRS microscopy, which requires samples to be viewed ex vivo, Raman spectroscopy has been adapted for use in the setting of handheld systems albeit with significantly increased artifact and reduced resolution. Jermyn et al. described the utilization of a commercially available handheld contact fiber optic probe for intraoperative Raman spectroscopy; they initially tested the system in 17 glioma patients, identifying cancer cells with an accuracy of 92%.^74^ Jermyn et al. then subsequently combined multiple label-free optical tools, including optical coherence tomography, Raman spectroscopy, intrinsic fluorescence spectroscopy, and diffuse reflectance spectroscopy to create an intraoperative system with the ability to detect brain, lung, colon, and skin cancers with high accuracy, sensitivity, and specificity.^75^

### Local Drug Delivery

Local drug delivery to a glioma has the potential of avoiding the blood-brain barrier and allowing for higher therapeutic dosing while reducing the risk of systemic toxicities. Indeed, systemic temozolomide treatment has been tied to increased immunosuppression in GBM patients.^76^ In addition, preclinical studies have demonstrated an antagonistic relationship between systemic chemotherapy and the efficacy of immunotherapies.^77,78^ As a result, the local delivery of therapeutics directly to a tumor is an attractive treatment modality. Carmustine wafers, a form of local chemotherapy, is perhaps the most well-known and validated locally delivered treatment for glioblastoma.^79^ However, in practice, local delivery of a novel drug or biologic can be difficult and inaccurate, potentially contributing to the variability in results of various clinical trials investigating locally delivered treatments.

Convection enhanced delivery (CED) involves the stereotactic placement of catheters directly into a region of interest within the brain and allows for the targeted local delivery of a treatment into a tumor. CED has been used to deliver a number of anti-tumor therapies, including chemotherapies, immunotherapies, and virotherapies.^80–82^ CED techniques continue to improve through improvements in catheter design, methods for chronic treatment delivery, and imaging and modeling of treatment delivery.^81^ As CED technology continues to develop it may play an important part in the delivery of treatments to aid with unresectable or microscopic areas of tumor.

### Intraoperative Radiotherapy

Intraoperative radiotherapy (IORT) involves the delivery of radiation therapy, frequently a single high dose, to a resection cavity immediately following resection.^83^ It can theoretically damage or destroy residual microscopic tumor cells on the resection cavity edge that are unable to be identified or resected. Additional potential advantages include the ability to direct the radiation to the resection cavity intraoperatively, potentially increasing accuracy, and the ability to deliver radiation immediately after resection, reducing the time between surgery and the start of radiation for patients. Historically, IORT results have not demonstrated a convincing survival benefit in GBM patient.^84^ However, more recently an international pooled analysis of 51 GBM patients treated with IORT in addition to standard of care by Sarria et al. suggested a 25% increased overall survival in treated patients at 3 years. Similarly, a subsequently published dose-escalation phase I/II trial of IORT by Giordano et al. involving 15 patients with GBM demonstrated the safety of IORT and integration into the neurosurgical operating room, with a treatment time of approximately 30 minutes, prompting a phase III study.^85^ Results of future studies assessing the efficacy of IORT are necessary to determine the utility of this technology in patients.

### Ablative Technologies

The most common ablative technology other than radiation therapy studied for gliomas is laser interstitial thermal therapy (LITT). LITT involves the interstitial transmission of laser light through fiberoptic wires into a target tissue. MRI-guidance and thermography are then utilized to track the resulting thermal damage that is induced. LITT has been primarily used for deep-seated, focal, smaller tumors or in patients who are otherwise poor surgical candidates. There is a paucity of prospective studies evaluating LITT. Current evidence suggests that LITT is relatively safe with a recent study of 58 LITT treatments in recurrent and newly diagnosed GBM suggesting a 30-day morbidity of 16%.^86^ Complications associated with LITT include hemorrhage, significant tumor edema, and neurological deficit.^87^ The clinical efficacy of LITT for gliomas is still unclear, but may be associated with ablative coverage of the lesion.^86–89^ Additional prospective studies are needed to fully elucidate the clinical role of LITT, including its potential ability to open the peritumoral blood-brain barrier and synergize with chemo- or immunotherapies.^90^

Focused ultrasound has also been investigated for the ablation of GBM in small studies with limited efficacy. A study of three patients with GBM who underwent focused ultrasound thermoablation demonstrated an inability to achieve thermal coagulation of the tumors.^91^ A subsequent case report examining the use of magnetic resonance-guided focused ultrasound (MRgFUS) to ablate a GBM demonstrated successful ablation, but only in a small percentage of the tumor.^92^ As a result, due to technical restraints, the direct ablation of most tumors by focused ultrasound may not be feasible. However, focused ultrasound may be used in the settings of sonodynamic ablation in which focused ultrasound is used to activate a sonosensitizer, leading to tumor cell death.^93^ This has been demonstrated in preclinical rat glioma models using 5-ALA as a sonosensitizer.^94^ Further investigation is needed to understand the efficacy and safety of sonodynamic ablation in humans.

### Intraoperative Confocal Microscopy

Confocal microscopy allows for the intraoperative histological imaging of cells and has been utilized in other surgical specialties to aid in oncologic diagnoses.^95^ Sanai et al. demonstrated the initial feasibility of intraoperative confocal microscopy for a variety of brain tumor types.^96^ Subsequent studies have demonstrated the potential promise of confocal microscopy for in vivo histopathological imaging of brain tumors and in combination with 5-ALA for the identification of low-grade gliomas.^97,98^ Pavlov et al. also demonstrated the identification and diagnosis of low- and high-grade gliomas in a small cohort of patients using probe-based confocal laser endomicroscopy in combination with fluorescein.^99^ Additional large studies are needed to determine the efficacy of this technology in improving EOR and providing an intraoperative tumor diagnosis.

### Virtual and Augmented Reality in the Operating Room

Both augmented reality (AR), the overlaying of digital elements over real-life structures, and virtual reality (VR), the complete immersion into a virtual environment, have been investigated in brain tumor surgery. Interestingly, these technologies have been used by both patients and surgeons. Until more recently, surgeons primarily used AR and VR for educational purposes.^100^ These technologies, however, have since continued to be used more frequently in preoperative planning and intraoperatively.^101^ While still in the early stages of evaluation, the use of intraoperative AR and VR has been suggested to aid in glioma resection.^101^ In a study of 134 glioma patients, Sun et al. compared the outcomes between patients whose surgeries had been carried out using AR and VR based on functional neuronavigation and iMRI to a control group using anatomic neuronavigation; they found that the use of intraoperative AR and VR improved EOR and functional postoperative outcomes, highlighting the promise of these technologies.^102^

AR and VR technologies have also been utilized by patients during awake mapping to expand the repertoire of brain functions that can be mapped accurately.^103^ This is highlighted by Mazerand et al. who reported the accuracy of VR in detecting visual field defects and the case of a patient with a parietotemporal GBM in which VR was utilized in combination with subcortical mapping to identify and preserve the optic radiations.^104^ Similar studies have used VR to map social cognition^105^ as well as language function.^106^ Overall, VR and AR technologies offer exciting avenues of future investigation in brain tumor surgery with the ability to improve EOR as well as enhance postoperative functional outcomes. Practical limitations of of AR and VR technologies include lack of familiarity their use and potentially high operating costs.

## Conclusion

Multiple novel technologies are in development to aid with the intraoperative identification and removal of tumor cells, while reducing patient morbidity (Table 1). The high value of EOR in glioma patients, means these technologies will continue to play a significant role in operating rooms. In the near future, surgeons will be using novel imaging technologies to better visualize tumors, while also receiving rapid and detailed information on the molecular composition of resected tissue. The additional real-time information available to surgeons during surgery will subsequently allow them to maximize the safety of their resections and choose personalized therapies to deliver to the resection cavity following resection. Broadly, further large randomized clinical trials are needed to evaluate the significance and role of a number of the technologies discussed in this review. In addition, consideration should be given to the costs and resources associated with various technologies. Nevertheless, this is an exciting time for neurosurgical oncology and should result in improved outcomes for patients with brain tumors.

**Table 1.** Novel Technologies for Intraoperative Tumor Control Categorized by Potential Benefit

**Extent of resection**	Intraoperative MRI
	Intraoperative ultrasound
	5-ALA
	5-ALA alternatives
	FLIM
	Intraoperative mapping
	Intraoperative flow cytometry
	Intraoperative mass spectrometry
	Exoscopes
	Stimulated Raman scattering microscopy
	Raman spectroscopy
	Virtual and augmented Reality
	Intraoperative confocal microscopy
**Functional outcomes**	Intraoperative mapping
	Intraoperative MRI
	Virtual and augmented reality
**Delivery of intraoperative adjuvant therapy**	Convection enhanced delivery
	Intraoperative radiotherapy
